# Association between aluminum and iron exposure in maternal blood and umbilical cord blood and congenital heart defects in children

**DOI:** 10.7717/peerj.16755

**Published:** 2024-01-22

**Authors:** Jing Li, Chunhua Zhang, Baohong Mao, Qian Liu, Yanxia Wang, Bin Yi, Qing Liu

**Affiliations:** 1 Department of Scientific Research Center, Gansu Provincial Maternity and Child-Care Hospital, Lanzhou, Gansu Provincial, China; 2 Laboratory Medicine Center, Lanzhou University Second Hospital, Lanzhou, Gansu Provincial, China; 3 Department of Neonatology, Gansu Provincial Maternity and Child-Care Hospital, Lanzhou, Gansu Provincial, China; 4 Department of Gynecology, Gansu Provincial Maternity and Child-Care Hospital, Lanzhou, Gansu Provincial, China

**Keywords:** Aluminum, Iron, Metal exposure, Congenital heart defects, Cord blood, Birth cohort, Interaction test

## Abstract

**Background:**

Congenital heart disease (CHDs) is the major cause of mortality from birth defects, affecting up to 1% of live births worldwide. However, the relationship between aluminum (Al) and iron (Fe) levels and the risk of CHDs has yielded inconsistent results.

**Methods:**

We conducted a pair-matched case–control study that included 97 CHDs and 194 non-CHDs to investigate the association and interaction between Al/Fe exposure and the risk of CHDs in a birth cohort study in Lanzhou, China.

**Results:**

Higher concentrations of cord blood Al were associated with a greater risk of total CHDs (aOR = 2.826, 95% CI [1.009–7.266]) and isolated CHDs (aOR = 10.713, 95% CI [1.017–112.851]) compared to the lowest Al level. Both in maternal blood and cord blood, a significant dose-effect was observed between Al level and total CHDs (Ptrend < 0.05), but a similar pattern was not observed for Fe. High Al in addition to high Fe appeared to elicit a stronger association with CHDs than both lowest tertile of Al and Fe level in umbilical cord blood, particularly for multiple CHDs, septal defects and patent ductus arteriosus.

**Conclusions:**

Our study suggests that exposure to Al during pregnancy (≥2,408 μg/L) is significantly associated with an increased risk of CHDs in offspring, especially septal defects, and that high levels of Al and Fe are strongly correlated with fetal heart development. Further research is needed to understand the underlying mechanisms.

## Introduction

Congenital heart defects (CHDs) are the leading type of congenital disabilities, affecting 1 in 100 individuals (Houyel & Meilhac, 2021). These defects are associated with a high incidence of neonatal mortality and disability worldwide (Khoshnood et al., 2012; Vecoli et al., 2014). While research has shown that both genetic and environmental factors contribute to CHDs (Wessels & Willems, 2010), the underlying cause of the majority of CHDs cases remains unknown. Epidemiological studies have suggested that prenatal exposure to environmental factors, such as heavy metals, may be linked to birth defects, including CHDs (Ibrahimou et al., 2017; Jenkins et al., 2007; Liu et al., 2009).

Aluminum (Al) is a most abundant element in human life, with a wide range of uses including cookware, utensils, food additives, pharmaceutical ingredients, and water purifying agents (Campbell et al., 2004; Kumar & Gill, 2009). However, Al is a toxic pollutant and has a prolonged biological half-life (7 years) in the human body (Rahimzadeh et al., 2022). The more Al is ingested, the more it accumulates in the body, leading to detrimental effects on human health. Studies have shown that Al can accumulate in the placenta, causing developmental damage in offspring (Golub & Domingo, 1998). Animal experiments (Cranmer et al., 1986; Fu et al., 2014; Malekshah, 2005; Paternain et al., 1988) have also demonstrated that excessive Al intake can lead to embryonic developmental toxicity, including brain malformation and skeletal hypoplasia. Al exposure has also been linked to intrauterine growth retardation, embryo death, and female infertility. Additionally, studies reported that female workers at Al smelters have a high incidence of congenital anomalies (Sakr et al., 2010), and higher hair Al concentrations have been associated with an increased risk of CHDs in babies (Liu et al., 2016). Furthermore, Al has been identified as a cardiac teratogen for chickens, with exposure leading to ventricular septal and ventricular myocardial defects (ElMazoudy & Bekhet, 2016).

Numerous studies have highlighted the importance of optimal for cardiovascular development (Anand & Gupta, 2018; McAlpine et al., 2019; Puri et al., 2020; Yang et al., 2017). Iron (Fe) is a crucial nutrient for embryonic development, but the findings on its impact vary across studies. Numerous studies have highlighted the importance of optimal for cardiovascular development (Andersen et al., 2006; Yang et al., 2017), as well as its role in embryonic development in animal models (Shaw et al., 2010; Toyokuni, 2002). However, some studies have suggested that Fe overload can lead to heart defects (Rodriguez et al., 2007; Wang et al., 2021), while others have identified maternal Fe deficiency as a risk factor for CHDs (Deugnier, 2003; Yang et al., 2020). Additionally, previous research has demonstrated that Fe can interfere with the protein binding of Al and induce oxidative stress (Greger & Sutherland, 1997), which may contribute to the cardiac structural abnormalities in humans.

Furthermore, to our knowledge, few studies have independently assessed the correlation between Al/Fe concentrations in maternal whole blood (MB) and umbilical cord blood (CB) and the risk of CHDs in children. In order to thoroughly investigate the relationship between maternal Al/Fe exposure and the risk of CHDs, we carried out prospective nested case-control research in Lanzhou, China.

## Materials and Methods

### Study design and CHDs definitions

**Figure 1:**
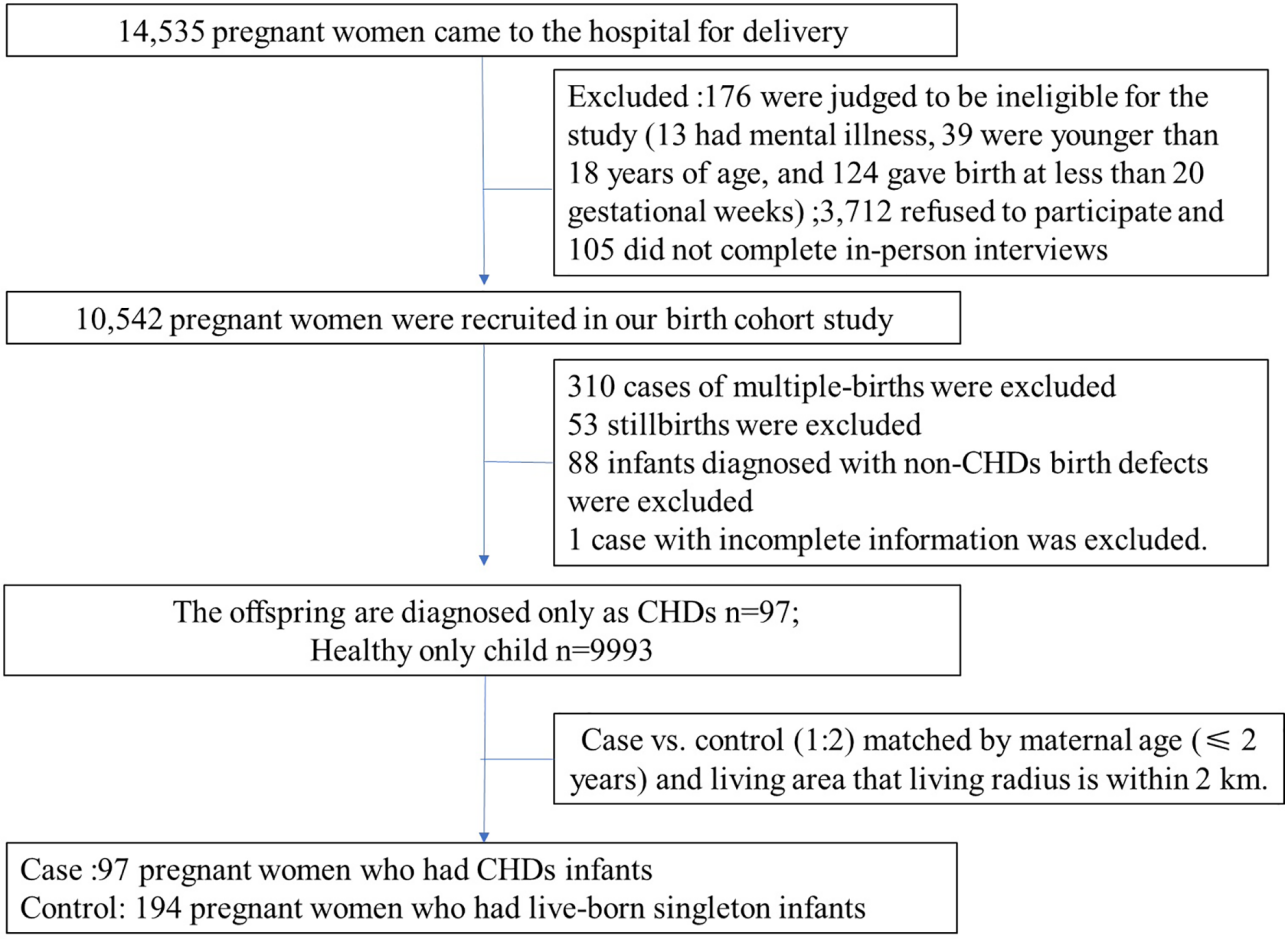
The flow chart of study population selection.

A total of 10,542 (response rates: 73.42%) birth cohort study was conducted (2010–2012) at the Gansu Provincial Maternity and Child Care Hospital in Lanzhou, northwest of China. Portions of this text were previously published as part of a preprint (Li et al., 2022). The study excluded individuals with multiple gestation, stillbirths, non-CHDs birth defects and the case with incomplete information, resulting in 10,090 live-born singleton participants. Data were collected as previously described in Li et al. (2021), Sun et al. (2022) (as showed in Fig. 1). Ethical approval was obtained from the Gansu Provincial Maternity and Child Care Hospital’s the Human Investigation Committees.

All the CHDs were classified into “isolated,” “multiple,” or “syndrome” based on the defect’s complexity of heart. “Isolated CHDs” refers to the case with either an isolated CHD; “Multiple CHDs” means the case with one more cardiac malformation, (*e.g*., atrial septal defect with patent ductus arteriosus) and “Syndrome defects” refer the infants with distinct CHDs associated with any non-CHD congenital anomalies. The cases were then further classified into three main subtypes based on anatomical structures: septal defects, atrial septal defect (ASD) and patent ductus arteriosus (PDA). Subtypes with insufficient cases were not discussed in our study. CHDs cases and non-CHDs controls were matched by maternal age (≤2 years) and living area that living radius is within 2 km. Among 10,090 individuals, a total of 97 mothers gave birth to live, singleton infants with CHDs (cases), and 194 selected matched mothers with healthy children (controls) were recruited. The cases were grouped into isolated defects (43 cases), multiple defects (46 cases), and syndrome defects (eight cases). More detailed information on the study population selection can be found in our previous studies (Huang et al., 2022; Mao et al., 2017; Sun et al., 2022).

### Al and Fe assessment

At the time of admission for labor, MB samples were collected from participating women and CB samples from newborns were collected immediately after delivery. These samples were then stored frozen at −80 °C in our hospital’s central laboratory until analysis. The detail in detection process of concentrations of ^27^Al and ^56^Fe by inductively coupled plasma mass spectrometry (ICP-MS; Themo Scientific, Bremen, Germany) were published in our previous studies (Huang et al., 2022; Sun et al., 2022). Quality control was ensured using Elements Whole Blood L-1 (No: 210113, purchased from Beijing Fubo Biotechnology Co., Ltd). The limits of detection (LOD) for Al and Fe in whole blood were both 1 μg/L, respectively, and when the concentration of them was below the LOD, it was reported as zero. The intra-assay and inter-assay coefficient of variation for Al were 4.68% and 4.85% respectively, and for Fe were 5% and 4.47% respectively.

### Statistical analysis

We compared the sociodemographic and lifestyle characteristics of individuals with CHDs and those without using χ^2^-test or Fisher’s exact test. Skewed continuous variables were analyzed using the Wilcoxon–Mann–Whitney U test and presented as median (range (min–max)). We also examined the distribution of Al and Fe levels between the CHDs and non-CHDs groups and assessed their inter-correlations. As there was no previous reference information available to determine the dose threshold of these metal elements, we categorized their concentrations into low (<25th percentile), medium (25th–75th percentile), and high levels (>75th percentile) based on their distribution among non-CHDs.

We used the Al/Fe levels of the CB and MB as predictors to investigate their association with CHDs and its subtypes. To adjust for confounding, we conducted conditional multivariable logistic regression and double-elements models that included both Al and Fe predictors. We adjusted for potential confounding variables, such as maternal hypertensive disorders during pregnancy, gestational diabetes, cesarean delivery, folic acid supplement, and average dietary energy intake during pregnancy. More detailed information about definition of variables can be found in our previous studies (Mao et al., 2017; Qiu et al., 2014). We also adjusted for smoking and drinking status and the gender of each neonate, which did not affect the multivariable adjusted estimates. Hence, our models did not adjust for additional covariates.

Herein, we employed categorical variables, as well as multiplicative and additive interaction, to investigate the modifying effects of Al/Fe on CHDs. To assess the analogous effects between Al and Fe, we categorized their levels into “low concentration group” (≤75th percentile) and “high concentration group” (>75th percentile), and analyzed each group’s effects, including low Al and low Fe, low Al and high Fe, high Al and low Fe, and high Al and high Fe. We also utilized multiplicative interaction and interaction contrast ratios (ICRs) to explore the possible interaction between Al and Fe on CHDs. All analyses were conducted using SAS 9.3 software (SAS Institute Inc., Cary, NC, USA), and statistical significance was determined by *P* < 0.05 or confidence intervals (95% CI) not including 1.0.

## Results

### Characteristics of participants

Table 1 displays the baseline demographic information of the maternal participants. Notably, there were significant differences in maternal hypertensive disorders during pregnancy, gestational diabetes and cesarean delivery between the two groups (*P* < 0.05), while no significant variations were observed in other maternal characteristics.

**Table 1:** Socio-demographic and lifestyle characteristics of pregnant women.

Characteristics		Controls (*n* = 194)	Cases (*n* = 97)	*P*-value
		*n* (%) or median ± SD or median (range)	*n* (%) or median ± SD or median (range)	
Maternal age (mean ± SD)		28.46 ± 4.33	29.54 ± 5.33	0.3045
Ethnic group				
	Han	189 (97.42)	90 (92.78)	0.0611
	Others	5 (2.58)	7 (7.22)	
BMI (kg/m^2^) (mean ± SD)		20.82 ± 2.73	21.29 ± 3.54	0.2645
	Missing	7	0	
Education, years				
	≤9	57 (29.84)	31 (32.98)	0.531
	10–15	69 (36.13)	34 (36.17)	
	≥16	65 (34.03)	29 (30.85)	
	Missing	3	3	
Smoking ^a^				
	No smoking	155 (79.9)	77 (79.38)	0.918
	Smoking	39 (20.1)	20 (20.62)	
Alcohol use				0.480
	No	193 (99.48)	97 (100)	
	Yes	1 (0.52)	0 (0)	
Employment status				
	No	91 (46.91)	47 (48.45)	0.8037
	Yes	103 (53.09)	50 (51.55)	
Sex of the child				0.480
	Boy	84 (43.52)	46 (47.92)	
	Girl	109 (56.48)	50 (52.08)	
Parity				
	Nulliparous	129 (66.49)	66 (68.04)	0.7918
	Parous	65 (33.51)	31 (31.96)	
Cesarean delivery				0.0002
	No	126 (65.97)	41 (42.71)	
	Yes	65 (34.03)	55 (57.29)	
	Missing	3	1	
Hypertensive disorders during pregnancy				
	No	194 (100)	87 (89.69)	<0.0001
	Yes	0 (0)	10 (10.31)	
Diabetes				
	No	194 (100)	91 (93.81)	0.0005
	Yes	0 (0)	6 (6.19)	
History of conception birth defect				
	No	193 (99.48)	96 (98.97)	0.6165
	Yes	1 (0.52)	1 (1.03)	
Family’s average monthly income (yuan)				
	≤3,000	107 (59.78)	57 (67.06)	0.2553
	>3,000	72 (40.22)	28 (32.94)	
	missing	15	12	
Average dietary energy intake during pregnancy (kcal)				0.7406
	<1,572.56	98 (50.52)	47 (48.45)	
	≥1,572.56	96 (49.48)	50 (51.55)	
Folic acid supplement				0.2148
	Nonusers	50 (25.77)	32 (32.99)	
	Users	144 (74.23)	65 (67.01)	
Al concentrations in MB (μg/L) (median (range))		2,117 (578–7,528)	2,450 (607–63,821)	0.0078
	<1,567	48 (24.74)	16 (16.49)	
	1,567–2,891	98 (50.52)	42 (43.30)	
	>2,891	48 (24.74)	39 (40.21)	
Al concentrations in CB (μg/L)		1,654 (104–8,238)	2.575 (453–24,424)	<0.0001
	<1,285	49 (25.26)	14 (14.43)	
	1,285–2,408	97 (50.00)	30 (30.93)	
	>2,408	48 (24.74)	53 (54.64)	
Fe concentrations in MB (μg/L) (median (range))		306,890 (17,450–834,780)	320,250 (61,720–928,640)	0.4801
	<228,973	49 (25.26)	20 (20.62)	
	228,973–379,056	96 (49.48)	51 (52.58)	
	>379,056	49 (25.26)	26 (26.80)	
Fe concentrations in CB (μg/L) (median (range))		431,090 (450–930,130)	500,700 (81,280–1312,090)	0.1125
	<336,272	48 (24.74)	17 (17.53)	
	336,272–530,785	97 (50.00)	49 (50.52)	
	≥530,785	49 (25.26)	31 (31.96)	

**Notes:**

a Maternal smoking including active smoking and passive smoking.

Abbreviation: BMI, Body mass index; MB, maternal blood; CB, cord blood.

**Figure 2:**
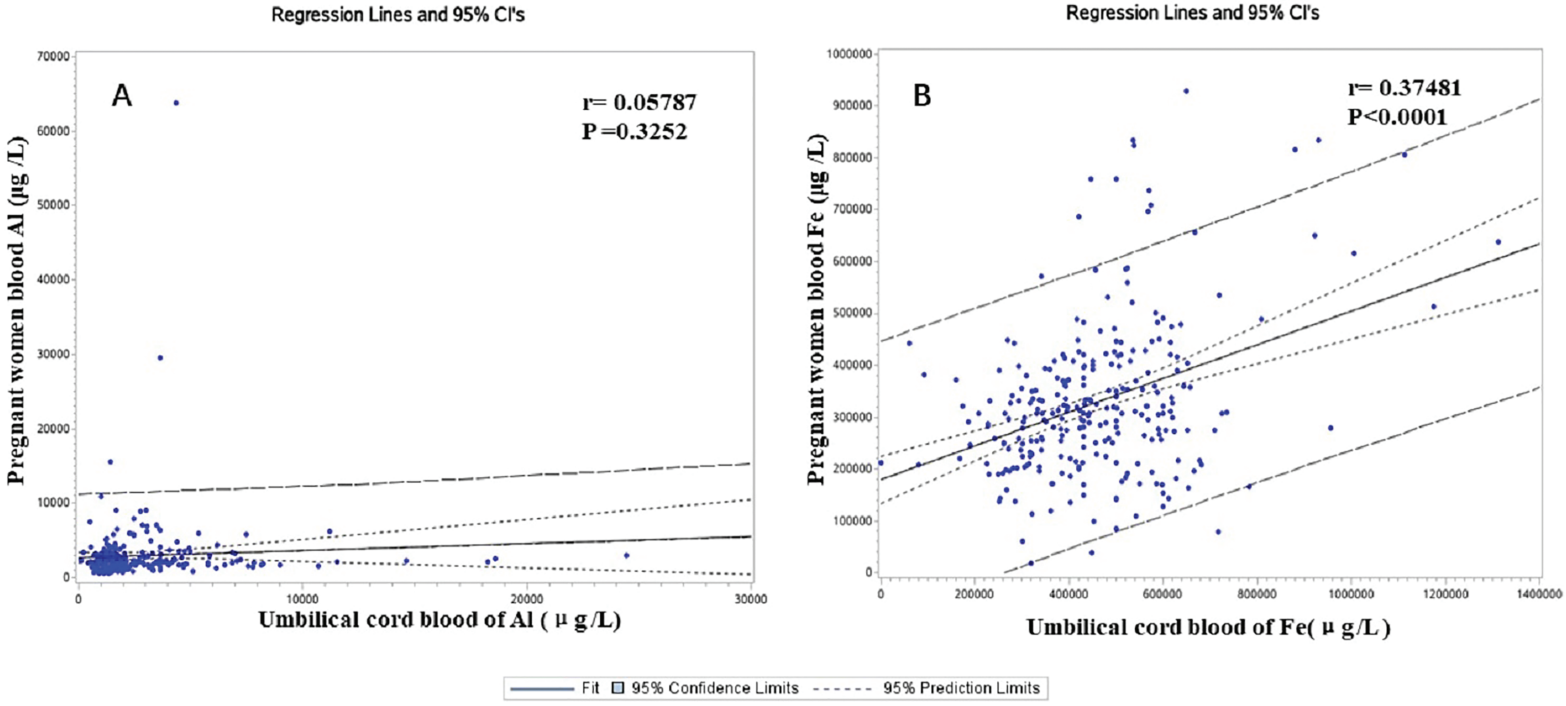
The correlation between the concentration of Al (A)/Fe (B) in pregnant women’s blood (MB) and that in umbilical cord blood (CB).

The Al’s median concentrations (range) of MB on non-CHDs and CHDs groups were 2,117 (578–7,528) and 2,450 (607–63,821) μg/L, respectively. Similarly, the Al’s median (range) CB’s concentrations were 1,654 (104–8,238) and 2,575 (453–24,424) μg/L in the case and control. Respectively, these results indicated that the Al concentration of MB and CB in the CHDs group was significantly higher than that in the control p (*P* < 0.001). The Fe levels in the MB and CB in the case group were not significantly different from those in the controls (*P* < 0.01). Fig. 2 shows that there was a positive correlation between the concentration of Fe in MB and that in CB (r = 0.37481, *P* < 0.0001). In contrast, no correlation was found between the concentration of Al in MB and that in CB (r = 0.05787, *P* = 0.3252).

### Conditional logistic regression model underling single-element

Initially, Al/Fe exposure and the risk of CHDs were examined separately using a multivariate single-element logistic regression model. The models were adjusted for various factors such as hypertensive disorders during pregnancy, diabetes, cesarean delivery, folic acid supplement and average dietary energy intake during pregnancy. Table 2 indicates that the correction of medium/high concentration of Al/Fe in MB was significant difference in CHDs and their subtypes compared to the low levels in this model. However, a significant dose-effect was observed between the Al level and total CHDs (*P* = 0.0274) and ASD in the MB (*P* = 0.0474).

**Table 2:** Associations between element levels in MB and congenital heart defects using multivariable single-element logistic regression models.

Elements	Low level		Middle level		High level		*P* for trend
	*n* cases/controls	aOR (95% CI)	*n* cases/controls	aOR (95% CI)	*n* cases/controls	aOR (95% CI)	
Al element							
Total CHD	16/48	ref.	42/98	1.415 [0.570–3.512]	39/48	1.831 [0.730–4.586]	0.0274
Multiple CHDs	9/48	ref.	20/98	0.988 [0.316–3.085]	17/48	1.108 [0.336–3.651]	0.0638
Isolated CHDs	4/48	ref.	20/98	3.790 [0.415–34.636]	19/48	6.311 [0.711–56.014]	0.1526
Septal defects	12/48	ref.	23/98	1.309 [0.466–3.679]	25/48	1.416 [0.494–4.055]	0.0701
ASD	10/48	ref.	19/98	1.176 [0.394–3.509]	19/48	1.589 [0.499–5.056]	0.0474
PDA	10/48	ref.	29/98	1.308 [0.453–3.771]	26/48	1.372 [0.449–4.195]	0.0859
Fe element							
Total CHD	20/49	ref.	51/96	1.083 [0.464–2.530]	26/49	1.275 [0.493–3.296]	0.3083
Multiple CHDs	10/49	ref.	22/96	1.055 [0.313–3.559]	14/49	1.587 [0.368–6.849]	0.1260
Isolated CHDs	7/49	ref.	25/96	3.025 [0.536–17.084]	11/49	1.731 [0.320–9.357]	0.9947
Septal defects	12/49	ref.	32/96	1.501 [0.528–4.265]	16/49	2.341 [0.703–7.791]	0.0948
ASD	10/49	ref.	26/96	1.450 [0.467–4.504]	12/49	2.017 [0.557–7.303]	0.1019
PDA	14/49	ref.	34/96	0.949 [0.340–2.648]	17/49	1.024 [0.317–3.303]	0.5583

**Note:**

MB, maternal blood; Al, aluminum; Fe, iron; CHDs, congenital heart defects; aOR, adjust odds ratio; CI, Confidence interval; PDA, Patent ductus arteriosus; ASD, atrial septal defect. A Low level serves as the reference group. Conditional logistic regression models were adjusted for hypertensive disorders during pregnancy, diabetes, cesarean delivery, folic acid supplement and average dietary energy intake during pregnancy.

Table 3 listed the associations between the element in CB and CHDs using multivariable single-element logistic regression models. When compared to the low quartiles (<25th) of Al, the difference was statistically significant in the high level of Al in CB in the total CHDs (OR = 3.037, 95% CI [1.205–7.655], *P* = 0.0091) and isolated CHDs (OR = 11.109, 95% CI [1.017–114.888]) when the confounders were adjusted. However, a similar pattern in the Al and Fe of CB was not found in other subtypes of CHDs. Nonetheless, statistical significance was detected in some subtypes of CHDs in Al, such as total CHDs (*P* = 0.0091), multiple CHDs (*P* = 0.0263), and PDA (*P* = 0.0263), as well as Fe level on total CHDs (*P* = 0.0127), multiple CHDs (*P* = 0.0315), and septal defects (*P* = 0.0201) for the trend.

**Table 3:** Associations between element in CB and congenital heart defects using multivariable single-element logistic regression models.

Elements	Low level		Middle level		High level		*P* for trend
	*n* cases/controls	aOR (95% CI)	*n* cases/controls	aOR (95% CI)	*n* cases/controls	aOR (95% CI)	
Al element							
Total CHD	14/49	ref.	30/97	0.861 [0.330–2.249]	53/48	3.037 [1.205–7.655]	0.0091
Multiple CHDs	10/49	ref.	14/97	0.419 [0.125–1.406]	22/48	2.055 [0.682–6.190]	0.0263
Isolated CHDs	4/49	ref.	14/97	4.405 [0.472–41.066]	25/48	11.109 [1.074–114.888]	0.1521
Septal defects	11/49	ref.	17/97	0.527 [0.176–1.574]	32/48	1.739 [0.629–4.809]	0.0596
ASD	10/49	ref.	15/97	0.572 [0.183–1.784]	23/48	1.587 [0.517–4.872]	0.1699
PDA	12/49	ref.	18/97	0.598 [0.190–1.889]	35/48	2.651 [0.893–7.868]	0.0335
Fe element							
Total CHD	17/48	ref.	49/97	0.730 [0.280–1.899]	31/49	1.619 [0.579–4.532]	0.0127
Multiple CHDs	10/48	ref.	21/97	0.775 (0.221–2.715]	15/49	2.317 [0.616–8.717]	0.0315
Isolated CHDs	5/48	ref.	23/97	0.560 [0.094–3.344]	15/49	0.728 [0.099–5.332]	0.4975
Septal defects	12/48	ref.	28/97	0.700 [0.217–2.255]	20/49	1.925 [0.564–6.571]	0.0201
ASD	11/48	ref.	22/97	0.457 [0.131–1.598]	15/49	1.040 [0.275–3.931]	0.1127
PDA	13/48	ref.	31/97	0.741 [0.247–2.220]	21/49	2.188 [0.625–7.657]	0.0625

**Note:**

CB, cord blood; Al, aluminum; Fe, iron; CHDs, congenital heart defects; aOR, adjust odds ratio; CI, Confidence interval; PDA, Patent ductus arteriosus; ASD, atrial septal defect. A Low level serves as the reference group. Conditional logistic regression models were adjusted for hypertensive disorders during pregnancy, diabetes, cesarean delivery, folic acid supplement and average dietary energy intake during pregnancy.

### Al/Fe exposure and CHDs risk using multivariable double-element logistic regression models

Subsequently, we conducted a multivariable double-elements logistic regression analysis (Table 4) to examine the impact of Al/Fe on CHDs. Our findings revealed that Al/Fe did not have a significant influence on other types. However, a dose-dependent effect was observed in MB for total CHDs (*P* = 0.0284) and PDA in Al (*P* = 0.0441).

**Table 4:** Associations between element levels in MB and congenital heart defects using multivariable double-elements logistic regression models.

Elements	Low level		Middle level		High level		*P* for trend
	*n* cases/controls	aOR (95% CI)	*n* cases/controls	aOR (95% CI)	*n* cases/controls	aOR (95% CI)	
Al element							
Total CHD	16/48	ref.	42/98	1.438 [0.569–3.633]	39/48	1.810 [0.700–4.678]	0.0284
Multiple CHDs	9/48	ref.	20/98	1.112 [0.335–3.692]	17/48	1.026 [0.296–3.558]	0.1774
Isolated CHDs	4/48	ref.	20/98	3.748 [0.372–37.811]	19/48	6.500 [0.562–75.193]	0.1291
Septal defects	12/48	ref.	23/98	1.338 [0.475–3.916]	25/48	1.016 [0.315–3.272]	0.2299
ASD	10/48	ref.	19/98	1.153 [0.370–3.591]	19/48	1.317 [0.374–4.636]	0.1996
Patent ductus arteriosus	10/48	ref.	29/98	1.338 [0.455–3.940]	26/48	1.400 [0.430–4.554]	0.0441
Fe element							
Total CHD	20/49	ref.	51/96	0.978 [0.412–2.319]	26/49	1.071 [0.387–2.963]	0.5652
Multiple CHDs	10/49	ref.	22/96	1.045 [0.303–3.601]	14/49	1.646 [0.303–3.601]	0.8834
Isolated CHDs	7/49	ref.	25/96	1.520 [0.253–9.120]	11/49	0.755 [0.091–6.245]	0.3537
Septal defects	12/49	ref.	32/96	1.466 [0.501–4.292]	16/49	2.685 [0.656–10.983)	0.7083
ASD	10/49	ref.	26/96	1.386 [0.436–4.405]	12/49	1.785 [0.419–7.617]	0.6458
Patent ductus arteriosus	14/49	ref.	34/96	0.886 [0.310–2.536]	17/49	0.949 [0.261–3.444]	0.1990

**Note:**

MB, maternal blood; Al, aluminum; Fe, iron; CHDs, congenital heart defects; aOR, adjust odds ratio; CI, Confidence interval; PDA, Patent ductus arteriosus; ASD, atrial septal defect. A Low level serves as the reference group. Conditional logistic regression models were adjusted for hypertensive disorders during pregnancy, diabetes, cesarean delivery, folic acid supplement and average dietary energy intake during pregnancy.

Table 5 presents the results of our analysis on the association between Al/Fe levels and CHDs in CB. Notably, significant differences were observed in the high quartiles of Al level compared to the low quartiles for total CHDs (OR = 2.826, 95% CI [1.009–7.266]) and isolated CHDs (OR = 10.713, 95% CI [1.017–112.851]) after adjusting for confounders. However, no similar pattern was observed for Fe levels in CB under this model. Nevertheless, a significant dose-effect was observed between the level of Al and total CHDs in CB (*P* = 0.0483).

**Table 5:** Associations between element in CB and congenital heart defects using multivariable double-elements logistic regression models.

	Low level		Middle level		High level		*P* for trend
	*n* cases/controls	aOR (95% CI)	*n* cases/controls	aOR (95% CI)	*n* cases/controls	aOR (95% CI)	
Al element							
Total CHD	14/49	ref.	30/97	0.857 [0.322–2.279]	53/48	2.826 [1.009–7.266]	0.0483
Multiple CHDs	10/49	ref.	14/97	0.343 [0.094–1.260]	22/48	1.679 [0.542–5.197]	0.1163
Isolated CHDs	4/49	ref.	14/97	4.219 [0.424–42.006]	25/48	10.713 [1.017–112.851]	0.1813
Septal defects	11/49	ref.	17/97	0.471 [0.147–1.507]	32/48	1.433 [0.484–4.240]	0.2665
ASD	10/49	ref.	15/97	0.606 [0.181–2.032]	23/48	1.541 [0.475–5.004]	0.3630
Patent ductus arteriosus	12/49	ref.	18/97	0.569 [0.171–1.896]	35/48	2.460 [0.815–7.427]	0.0980
Fe element							
Total CHD	17/48	ref.	49/97	0.701 [0.257–1.912]	31/49	1.206 [0.413–3.526]	1.1411
Multiple CHDs	10/48	ref.	21/97	0.784 [0.221–2.789]	15/49	2.187 [0.550–8.694]	0.2216
Isolated CHDs	5/48	ref.	23/97	0.655 [0.063–4.711]	15/49	0.655 [0.053–8.052]	0.9215
Septal defects	12/48	ref.	28/97	0.748 [0.226–2.477]	20/49	1.725 [0.474–6.277]	0.1246
ASD	11/48	ref.	22/97	0.521 [0.141–1.924]	15/49	1.021 [0.251–4.154]	0.2965
Patent ductus arteriosus	13/48	ref.	31/97	0.769 [0.246–2.405]	21/49	2.011 [0.554–7.301]	0.2785

**Note:**

CB, cord blood; Al, aluminum; Fe, iron; CHDs, congenital heart defects; aOR, adjust odds ratio; CI, Confidence interval; PDA, Patent ductus arteriosus; ASD, atrial septal defect. A Low level serves as the reference group. Conditional logistic regression models were adjusted for hypertensive disorders during pregnancy, diabetes, cesarean delivery, folic acid supplement and average dietary energy intake during pregnancy.

### Interaction effects between Al and Fe

Furthermore, we investigated the potential interaction effects between MB and CB levels of Al and Fe to determine whether their combined was synergistic, additive or antagonistic in relation to the incidence of CHDs. We dichotomized the levels as “low” (≤75th percentile) and “high” (>75th percentile), and included cross-product terms in a multivariable logistic regression model, adjusted for confounders as previously described (Table 6). We found no significant between correlation categorical variables in CHDs and all subtypes in MB. However, in CB, high levels of both Al and Fe were strongly positively associated with offspring’s cardiac development compared to high levels of Al alone in total CHDs (aOR = 5.273, 95% CI [1.897–14.657]), multiple CHDs (aOR = 7.820, 95% CI [1.672–36.588]), septal defects (aOR = 4.747, 95% CI [1.428–15.780]), and PDA (aOR = 13.147, 95% CI [2.415–71.565]). However, we did not detect any significant multiplicative or additive interactions between Al and Fe in both MB and CB (see Table 6).

**Table 6:** Interaction effects among blood AL and Fe on the odds for total congenital heart defects using multivariable logistic regression.

Exposure	Total CHDs		Multiple CHDs		Isolated CHDs		Septal defects		ASD		PDA	
	n cases/controls	aOR (95% CI)	*n* cases/controls	aOR (95% CI)	*n* cases/controls	aOR (95% CI)	*n* cases/controls	aOR (95% CI)	*n* cases/controls	aOR (95% CI)	*n* cases/controls	aOR (95% CI)
MB												
Low Al and low Fe	50/117	Ref.	24/117	Ref.	22/117	Ref.	31/117	Ref.	26/117	Ref.	20/117	Ref.
Low Al and high Fe	8/29	0.759 [0.210–2.737]	5/29	2.023 [0.390–10.492]	2/29	NA	4/29	1.361 [0.249– 7.428]	3/29	1.014 [0.155–6.635]	3/29	0.872[0.183–4.155]
High Al and low Fe	21/28	1.202 [0.524–2.758]	8/28	1.116 [0.349–3.572]	10/28	1.286 [0.304–5.445]	13/28	0.763 [0.256–2.278]	10/28	1.094 [0.335–3.570]	12/28	1.045[0.358–3.052]
High Al and high Fe	18/20	1.606 [0.679–3.797]	9/20	1.381 [0.435–4.383]	9/20	2.355 [0.482–11.502]	12/20	1.737 [0.659–4.579]	9/20	1.751 [0.604–5.079]	14/20	1.185[0.419–3.349]
Cross-Product term for interaction		1.27 [0.671–2.244]		1.083 [0.505–2.317]		1.100 [0.259–4.681]		1.163 [0.570–2.375]		1.247 [0.545–2.852]		1.023[0.487–2.148]
Interaction on the additive scale (RERI)		0.631 [−0.469–1.731]		0.572 [−1.180–2.324]		1.220 [−0.715 to 3.156]		1.159 [−0.326–2.644]		1.471 [−0.270 to 3.213]		0.870[−0.371 to 2.112]
CB												
Low Al and low Fe	32/113	Ref.	33/113	Ref.	13/113	Ref.	20/113	Ref.	18/113	Ref.	21/113	Ref.
Low Al and high Fe	12/33	1.121 [0.353–3.563]	7/33	1.175 [0.232–5.934]	5/33	0.663 [0.099–4.429]	8/33	1.170 [0.283–4.840]	7/33	0.976 [0.219–4.350]	9/33	1.134[0.248–5.182]
High Al and low Fe	32/34	2.527 [1.063–6.012]	14/32	2.183 [0.709–6.719]	15/32	2.405 [0.508–11.393]	20/32	1.719 [0.616–4.801]	15/32	1.656 [0.547–5.011]	23/32	2.202[0.767–6.323]
High Al and high Fe	19/16	5.273 [1.897–14.657]	8/16	7.820 [1.672–36.588]	10/16	3.290 [0.646–16.765]	12/16	4.747 [1.428–15.780]	8/16	3.751 [0.926–15.187]	12/16	13.147[2.415–71.565]
Cross-Product term for interaction		1.039 [0.818–1.319]		0.862 [0.616–1.206]		1.298 [0.803–2.098]		0.893 [0.672–1.186]		0.837 [0.601–1.164]		1.079[0.796–1.462]
Interaction on the additive scale (RERI)		0.754 [−1.022 to 2.510]		1.238 [−0.1354 to 3.829]		1.825 [−1.319 to 4.970]		1.148 [−0.951 to 3.247]		0.717 [−1.500 to 2.934]		0.451[−1.593 to 2.495]

**Note:**

MB, maternal blood; CB, cord blood; Al, aluminum; Fe, iron; CHDs, congenital heart defects; aOR, adjust odds ratio; CI, Confidence interval; PDA, Patent ductus arteriosus; ASD, atrial septal defect. A Low level serves as the reference group. Conditional logistic regression models were adjusted for hypertensive disorders during pregnancy, diabetes, cesarean delivery, folic acid supplement and average dietary energy intake during pregnancy.

## Discussion

Our study aimed to identify the potential interaction between maternal exposure to Al and Fe during pregnancy and CHDs in children, measured in MB and CB simultaneously. Our results showed that the level of Al was higher in the CHDs groups than that in the controls (Table 1). Logistic regression analysis revealed a significant association between maternal exposure to Al and the risk of CHDs in children, with higher concentrations of Al associated with an increased risk of CHDs. This association was consistent across CHDs subtypes, including septal defects, PDA, and isolated CHDs in CB (see Tables 4 and 5). Finally, our findings suggested that mothers with high levels of both Al and Fe had a higher risk of CHDs compared to mothers with low levels of both (see Table 6).

### Associations between Al and CHDs

Al is the third most abundant element in the Earth’s crust, but its environmental pollution is widespread due to its extensive use in various industries and products, such as cookware, utensils, toys, and medical treatments (Liu et al., 2018; Ward, Zhang & Crichton, 2001). However, exposure to Al can be hazardous to human health, as it can accumulate in the body and cause teratogenic, carcinogenic, and mutagenic effects. Despite this, little is known about the potential risk of maternal aluminum exposure on congenital disabilities, particularly congenital heart defects (CHDs). Previous research (Greger & Sutherland, 1997) has shown that a healthy person may accumulate 30–50 mg/kg of Al, with normal serum levels ranging from 1–3 μg/L (Wiesner, Gajewska & Paśko, 2021). However, our recent findings indicate that the median Al levels in MB (2,117 μg/L) were higher than those in a normal person, while the median levels in CB (1,654 μg/L) were significantly lower than those, suggesting that the placenta may act as a physical barrier against metallic elements. Animal studies (ElMazoudy & Bekhet, 2016) have shown that Al is teratogenic to the nervous system and can affect the development of blood vessels, the heart, and neurons. While a study in India (Shekhawat et al., 2023) found a correlation between maternal and cord serum Al levels, our study did not find a significant correlation between the concentration of Al in MB and CB. Moreover, the mean concentration of maternal and cord serum Al in our study was higher than that in India suggesting that the placenta may not be an effective barrier against Al carcinogens at higher levels.

The development of the heart tube is affected by Al toxicity, which sheds light on the impact of Al on human embryonic heart development. Previous studies in China (Liu et al., 2018; Liu et al., 2016) analyzed hair samples using ICP-MS and found a significant difference in hair Al content and the occurrence of total CHDs in the offspring (aOR = 2.32, 95% CI [1.72–3.13]). This suggests that Al is involved in CHDs through oxidative stress. A Polish study also showed that high levels of Al in MB (mean ± SD: 250.3 ± 176.2 µg/L) and amniotic fluid (144.8 ± 54.1 µg/L) are associated with birth defects. Moreover, a high level of Al is the best predictor of birth defects (β coefficient = −0.28; *P* = 0.02) (Kocylowski et al., 2019). Our recent study and other accumulating evidence indicate that high levels of Al in MB significantly increase the risk of CHDs in offspring, as seen in hair, amniotic fluid, or CB. Notably, the risk of Al exposure is related to fetal CHDs occurrence in a dose-dependent manner with elevated Al levels (≥2,408 μg/L in the CB), supporting the theory that maternal Al exposure increases the risk of CHDs in children.

### Associations between Fe and CHDs

On the other hand, Fe has been extensively studied for its role in the occurrence of CHDs. Fe plays a crucial role in the growth and development of the embryo in many animal studies (Harris & Messori, 2002; Kell, 2009; Toyokuni, 2002), particularly in cardiovascular development (Anand & Gupta, 2018). However, the molecular mechanisms underlying this relationship are not well-defined and evidence on the correlations between Fe nutritional status and CHDs among humans has been controversial in the past decade.

Several studies have suggested that maternal Fe deficiency is a risk factor for CHDs (Deugnier, 2003; Yang et al., 2020). Additionally, low ferritin levels may affect the utilization of folate, even when intake is adequate, by interacting with other nutrients such as folate (O’Connor, 1991). A meta-analysis study has indicated that maternal folate deficiency may reduce the risk of CHDs (Feng et al., 2015). Our previous study found that pregnant women who did not take a folate supplement and had low folic acid intake from their diet had two-fold increase in CHDs rates in their offspring (Mao et al., 2017). Therefore, in our current study, we considered pregnant women’s folate intake as a confounder factor. Iron-deficient women of childbearing age have high iron demands, and pregnant women are more susceptible to iron deficiency. The may lead to tissue hypoxia and stress response, which can affect fetal cardiac development (Fisher & Burggren, 2007). However, recent studies have shown that Fe overload can also cause heart defects (Wang et al., 2021). To our knowledge, only one study in China has explored he correlation between maternal Fe nutritional status and the rate of offspring’s CHDs (Yang et al., 2020). Our study classifies the exposure of Fe status in both MB and CB directly and found that the association of high Fe concentration did not differ statistically in CHDs and their subtypes, even after adjusting for confounders. The mechanisms underlying these associations between Fe deficiency and the development remain unclear and require further investigation.

### Interactions between trace elements

To the best of our knowledge, previous studies have not thoroughly investigated the interactions between the trace Al and Fe elements on CHDs. However, this lack of research may be due to small sample size. Given the limited research on the co-exposed effects of mental elements on fetal heart development, further studies are necessary to understand fetal susceptibility to environmental exposure. Our study found no significant differences between categorical variables in CHDs and subtypes in MB. However, in CB, high levels of both Al and Fe exist a stronger impact on fetal heart development than high levels of Al alone in total CHDs, multiple CHDs, septal defects and PDA. The potential pathogenic mechanisms of Al-induced CHDs include the accumulation of Al in lysosomes, which damages cardiomyocytes, the disruption of transferrin synthesis, leading to increased free Fe levels (Chen, Zeng & Hu, 2010; Day et al., 1991; Harris & Messori, 2002; Hémadi et al., 2003) and abnormal ROS activation (Gorsky et al., 1979; Kell, 2009; Oteiza, 1994), and the interference with cytoskeleton polymerization through binding to nuclear chromatin (Zhang et al., 2016). Our study also suggests that simultaneous exposure to high levels of Fe may enhance the effects of Al exposure.

The present study offers several advantages. Firstly, we identified maternal and fetal biomarkers during pregnancy, and collected demographic, pregnancy, and birth data from clinical records, which minimizes the risk of recall bias. Secondly, we included age-matched controls and ensured that their residential area was within a 2 km radius, ensuring that our sampling framework was representative. Thirdly, we evaluated the correlation between Al/Fe in both MB and CB and their association with CHDs, adjusting for potential confounding variables. However, the present study has some limitations. The small sample size limits the statistical power of our association and interaction studies of CHDs subtypes. Additionally, exposure misclassification may have occurred as MB was collected during the third trimester, which may not reflect the association between early pregnancy exposure and CHDs. Despite these limitations, our findings suggest a possible pathogenic role of Al in CHDs and a synergistic effect with Fe in pathogenicity, which requires further investigation to confirm. It should be noted that our cohort may not be representative of pregnant women in China due to the small sample size, but the prevalence of CHDs in our population (9.3% live births) was similar to that reported in other studies in China (Zhao et al., 2021).

## Conclusions

Our study revealed a significant link between fetal exposure to high levels of Al (≥2,408 μg/L) in CB and an elevated risk of offspring in CHDs. Notably, we also discovered that concurrent exposure to high levels of Fe may exacerbate the negative impact of Al on cardiac development. These findings, in conjunction with fetal echocardiography, can aid in identifying high-risk pregnancies with Al contamination in Gansu Province and reducing the incidence of birth defects.

## Supplemental Information

10.7717/peerj.16755/supp-1 Supplemental Information 1 Data.

## Additional Information and Declarations

### Data Availability

The following information was supplied regarding data availability: The raw measurements are available in the Supplemental Files.
